# Exploring perceptions of the services offered in Tanzanian sober houses: a mixed- methods study among service users and providers

**DOI:** 10.1186/s12913-025-12384-7

**Published:** 2025-02-14

**Authors:** Samuel Janson, Stella E. Mushy, Mecca McPherson, Frank Mhando, Larissa Jennings Mayo-Wilson, Masunga K. Iseselo, Haneefa Saleem, Jerome Kamwela, Jumanne Issango, Justin Knox, Gaspar Mbita, Deng B. Madut, Jan Ostermann, Nathan Thielman, Betuel Mwasa, Donaldson F. Conserve, BRIDGE Africa

**Affiliations:** 1 The George Washington University Milken Institute School of Public Health, Washington, DC USA; 2 Muhimbili University of Health and Allied Sciences, Dar Es Salaam, Tanzania; 3 Temple University College of Public Health, Philadelphia, PA USA; 4 University of Johannesburg, Johannesburg, South Africa; 5 The University of North Carolina Gillings School of Global Public Health, Chapel Hill, NC USA; 6 The Johns Hopkins University Bloomberg School of Public Health, Baltimore, MD USA; 7 Tanzania Commission for AIDS (TACAIDS), Dar Es Salaam, Tanzania; 8 Columbia University Irving Medical Center, New York, NY USA; 9 New York State Psychiatric Institute, New York, NY USA; 10 Columbia University Mailman School of Public Health, New York, NY USA; 11 Jhpiego, Monrovia, Liberia; 12 Duke University School of Medicine, Durham, NC USA; 13 University of South Carolina, Columbia, SC USA; 14 Benjamin Mkapa Foundation, Dar Es Salaam, Tanzania

**Keywords:** Sober houses, Substance use treatment, People who use drugs, Tanzania, Residential treatment, HIV

## Abstract

**Background:**

In Tanzania, residential treatment centers for alcohol and other drugs, locally known as “sober houses,” play a critical role in the treatment of people living with a substance use disorder (SUD), but little is known about the services they offer and service users’ and providers’ perceptions of those services. We aimed to address these gaps in knowledge and to better understand where evidence-based interventions may be able to address gaps in service provision.

**Materials and methods:**

This study used a mixed-methods approach across four sober houses in Dar es Salaam, Tanzania. We conducted 48 semi-structured interviews with a sub-sample of sober house service users (*n* = 38) and service providers (*n* = 10). Eighty-six (86) service users also completed a written survey to capture demographic information and assess knowledge of HIV and willingness to use HIV preventive care. All interviews were audio-recorded, translated into English, and then coded according to constructs developed with the Recovery Capital Framework. Following coding, a thematic analysis was conducted for the qualitative data using the framework developed by Braun and Clarke.

**Results:**

Service users were generally positive about the treatment they were receiving but identified gaps in health service provision related to HIV, as well as a lack of preparation to address the employment-related challenges they face in the community after completing treatment. Service providers largely agreed with users’ perceptions of needs and identified a lack of clinical personnel in sober houses and funding challenges as barriers to meeting these needs.

**Conclusion:**

Sober houses provide treatment services to Tanzanians with SUD that service users largely view as positive. This evaluation identified employment challenges after treatment completion and gaps related to HIV care in the sober house. Further research is needed to investigate how interventions can be adapted to the sober house setting to meet these needs.

**Supplementary Information:**

The online version contains supplementary material available at 10.1186/s12913-025-12384-7.

## Introduction

In Tanzania, harmful substance use and substance use disorders (SUD) pose major threats to the population health and economic development of the country [1–4]. Tanzania has significantly higher levels of yearly per capita alcohol consumption than its regional neighbors at 11.27 L per capita compared to 2.78 L in Kenya and 2.3 L in Mozambique [5], though the actual national prevalence of alcohol use disorder (AUD) is unknown [6, 7]. Data concerning drug use is less available, but attempts to map hotspots of substance use throughout the country have identified Tanga, Mwanza, and Dar es Salaam as areas with comparatively high levels of illicit drug use, in part due to their position on known drug trafficking routes [1, 3, 8, 9].

The Government of Tanzania (GoT) has taken steps to expand access to treatment for SUD in the country. In 2011, Tanzania became the first sub-Saharan African (SSA) country to approve methadone administered through specialized clinics as a treatment option for people with opioid use disorder (OUD) [10]. As of 2022, 13,696 people (95.5% male, 4.5% female) were enrolled in methadone clinics across the country [4]. In addition to methadone clinics, psychiatric hospitals in Tanzania are a substantial source of treatment for SUD. In 2022, there were over 850,000 admissions across the country for SUD treatment in psychiatric hospitals (51% male, 49% female) [4].

Long-term, residential treatment centers are an additional source of treatment for Tanzanians with a SUD. In 2009, the first residential treatment center in Tanzania, locally known as a “sober house,” was opened in Zanzibar to meet the growing need for OUD treatment [11]. Since 2009, the volume of sober houses continued to grow in Tanzania, reaching 43 registered facilities in 2022. Sober house treatment is paid for out-of-pocket, and the treatment duration varies between one month to over one year [4]. These facilities are often located in the communities surrounding large urban centers, as the guidelines from the Drug Control and Enforcement Authority (DCEA) require that sober houses not operate in busy, urban environments [4].

Sober houses are highly utilized. Between 2019 and 2022, there were over 12,000 treatment admissions to sober houses in Tanzania, almost all of which (96.5%) were men [4]. Studies from Tanzania show that societal stigma towards substance use is higher towards women than men, which may inhibit treatment seeking by women [12–14]. Moreover, gender parity is much closer for SUD treatment in psychiatric hospitals, which may indicate a preference by women for these settings or simply be the result of few operational sober houses for women [4].

The treatment of Tanzanians with SUD is also integral to national efforts to control diseases commonly associated with substance use, including HIV [15]. The National AIDS Control Program of Tanzania (NACP) has recognized people who use drugs (PWUD) and people who inject drugs (PWID) as key vulnerable populations in control of the HIV epidemic, with estimated seroprevalence rates of 36% and 20%, respectively, higher than the national prevalence rate of 4.5% [15]. In addition to the association of new HIV cases with substance use, untreated SUD is also associated with poorer treatment outcomes, including accelerated disease progression and lower adherence to antiretroviral therapy (ART) [9, 16, 17]. Given the high incidence of HIV among PWID in Tanzania, this population stands to benefit immensely from HIV preventative treatment, namely oral pre-exposure prophylaxis (PrEP). However, national uptake of PrEP has been slow, and additional strategies are needed to reach PWUD [18].

Despite the essential role that sober houses play as a treatment option for individuals with a SUD in Tanzania, there has not been a comprehensive exploration of the perceptions of service users and providers regarding the treatment offered in sober houses and of areas for service improvement. One of the only published studies conducted in Tanzanian sober houses is a 10-year follow-up study among service users who received treatment in Tanzania’s first sober house (“Detroit House”) during its first year of operation, all of whom were treated for OUD [11]. This study found that 85% of participants (*n* = 89) reported being “drug-free” at the 10-year follow-up, and 55% of participants were receiving ongoing methadone treatment [11]. However, an additional qualitative study conducted among sober house service users found that substance use recurrence following treatment is common, and that easy access to drugs, community stigma, idleness, and peer influence all challenge long-term abstinence from drugs and alcohol [19].

The aim of our study was to build on these findings, guided by three questions: 1.) What services are sober houses currently providing?; 2.) Are these services preparing service users with the skills and resources needed to remain abstinent from drugs and alcohol after treatment?; and 3.) What needs of service users may be addressed through the implementation of an evidence-based intervention in sober houses? We asked these questions, to assess the possibility of an interventional study and were guided by the first step of the ADAPT-ITT model, *assessment*. The ADAPT-ITT model is a useful tool for assessing needs and planning for the adaptation and eventual implementation of evidence-based interventions across contexts [20].

## Methods

### Study design

This study used a mixed-methods approach, operationalized through a convergent mixed-research design, with sober house service users (*n* = 38) and providers (*n* = 10) across four sober houses in Dar es Salaam. Semi-structured interviews were conducted in Kiswahili in a private space at the sober house site. Interviews lasted between 30 and 90 min and all were audio recorded. The interview guides were created for this study and are available as Supplementary Files 2 and 3. A short, written survey was provided to consenting service users (*n* = 86), which collected demographic information (age, place of residence, age of substance use initiation, etc.) and a basic health history (HIV testing history, diagnoses of mental illness, awareness and use of PrEP, etc.). The survey was created for this study and is available as Supplementary File 1.

### Study setting

The geographic area for this study, Dar es Salaam, Tanzania, was chosen for two primary reasons. First, there is a high concentration of sober houses in the city and surrounding area. Of the 43 registered sober houses in 2022 across Tanzania, 15 were in Dar es Salaam [4]. Second, Dar es Salaam and the surrounding Pwani region have been identified as having one of the highest rates of substance use in the country [1, 3]. For these reasons, Dar es Salaam was identified as a hotspot of both treatment demand and sober house availability, presenting an ideal setting for this study.

### Study population and recruitment

Sober house sites were recruited through convenience sampling. Members of the study team based in Tanzania leveraged personal contacts to begin compiling a list of sober houses in Dar es Salaam and their associated contact information. After contacting the administrators at each of these houses, we identified four sober houses that were eligible for participation. The criteria for eligibility were: 1.) Located in Dar es Salaam or the surrounding area; 2.) Registration with the Drug Control Enforcement Authority (DCEA); 3.) Operational for at least one year and; 4.) Willingness of management to allow a site visit by the study team lasting at least two days. Of the four sober houses in our sample, three treated male service users and one treated female service users. This is consistent with sober house admissions nationally which are overwhelmingly male (96.5%). The patient population of the four sober houses recruited into the study varied considerably, ranging from 8–44 clients, with a combined total of 117 service users across houses. To be eligible for participation in the study, service users had to be: 1.) 18 years or older; 2.) Able to provide informed consent; and 3.) Not currently undergoing detoxification from drugs or alcohol. The eligibility criteria for sober house service providers were: 1.) 18 years or older; 2.) Able to provide informed consent; and 3.) Have been employed in the sober house for at least one month. All service users were invited to participate in the brief survey and semi-structure interview. Sober house service providers were only invited to participate in an interview. Service providers were recruited from a variety of roles including house managers, psychological counselors (holding bachelor’s and master’s degrees), and peer counselors.

## Data collection process

Semi-structured interviews were led by trained data collectors, fluent in Kiswahili and English, with qualitative data collection experience. Data collectors were paid and signed a confidentiality agreement prior to data collection. The study was first explained to all sober house service users together, and then any individual who was interested in participating in an interview could approach a data collector to participate. Service providers participated in interviews based on their availability. No compensation was provided to service users for their participation but the research team donated items such as food and exercise equipment to the sober houses that all could access. Service providers were given a per diem during data collection days equivalent to 15 USD. Interviews were conducted one-on-one, and participants provided written informed consent prior to starting the interview which included consent to be audio recorded. Interviews were conducted using two unique interview guides for service users and providers. During the interviews, service users were asked questions regarding their life before entering the sober house, motivations to seek treatment, perceptions of the treatment they are receiving in the sober house, knowledge and uptake of various health services including HIV care, plan to maintain substance use abstinence after completing treatment, and unmet needs in the sober house. Providers were asked about their educational backgrounds, the intake process for new clients in the sober house, the range of medical and psychosocial services offered in the facility, ways they monitor quality and patient outcomes, and unmet needs of service users and providers.

Furthermore, service users were also invited to participate in a 25-question survey. The written survey was meant to provide further context to the qualitative interviews and was completed on paper. The questions were read aloud to participants who were given the opportunity to ask clarifying questions. The same eligibility criteria for interviews were applied to the written surveys, and written informed consent was again gathered. Surveys captured demographic information such as age, place of residence, tribe, religion, prior sober house treatments, and duration of stay in the sober house. Participants were also asked questions about their knowledge of and willingness to use HIV self-test kits (HIVST) and pre-exposure prophylaxis (PrEP). These questions were included in the survey questionnaire given the high comorbidity of SUD and HIV and Tanzania’s national strategy to increase HIV testing through HIVST [9, 21–23]. Service users were first asked if they had ever heard of HIVST or PrEP, and then received a brief, live demonstration of how to use an HIVST kit, as well as an explanation of what PrEP is and how it is used. The demonstration and accompanying information were to ensure participants had adequate information to answer the related survey questions about their willingness to use them if made available in the sober house.

### Data analysis

Following the completion of data collection, all interviews were transcribed in Kiswahili and translated into English. Transcripts then underwent qualitative coding using NVivo 14 (Lumivero, 2023) by two coders working independently, who later compared coded transcripts for agreement. Passages that were coded differently were discussed between coders. When the two coders could not agree, a third study team member made the final decision on how to code select passages.

Prior to coding, a qualitative codebook was developed, guided in part by the Recovery Capital Framework (RCF) [24, 25]. The term “recovery capital” refers to the internal and external resources that assist people with a SUD achieve long-term abstinence from drugs and alcohol. Recovery capital is broken down into three main constructs. The first construct is *personal recovery capital,* which encompasses factors such as good health and the availability of adequate material resources. The concept of *personal recovery capital* was used to create codes for responses related to HIV status, testing history, medical services received in the sober house, employment prospects outside of the sober house, and stable housing. Another construct in the RCF is *social recovery capital,* which emphasizes relationships that are supportive to an individual’s recovery. This construct was important for creating codes related to whether sober houses promote positive peer and mentoring relationships among service users and providers, and the presence of supportive familial and social relationships outside of the sober house. The third construct, *community recovery capital,* highlights the role of available services within the community, as well as attitudes in the community that can both help and hinder recovery. Community recovery capital was used to generate codes related to service users’ plans for remaining abstinent from drugs and alcohol, the availability of 12-step meetings, access to adequate medical services, and experiences with stigma or discrimination related to their substance use.

The RCF was appropriate for analysis of sober house interviews as its constructs could be used for investigating strengths (existing recovery capital) as well as needs (deficiencies in recovery capital). After coding, study team members reviewed the coded transcripts and performed a thematic analysis using the method established by Braun and Clark to identify any latent themes not originally included in the codebook [26]. From this process, several themes were generated, and additional codes were developed to encompass those themes. We grouped responses by whether they were provided by a service user or provider to analyze the congruency of perspectives between the two groups and to look for points of distinction.

Survey responses were transferred from paper surveys to Qualtrics^TM^ for analysis (Qualtrics, Provo UT). Qualtrics was used for generating descriptive statistics from survey responses.

### Data quality

The trustworthiness of the data was evaluated using Guba and Lincoln’s guidelines for naturalistic inquiry [27]. These guidelines were created to evaluate the trustworthiness of qualitative data from social and behavioral research and the authors posit four components of trustworthiness that should be evaluated: credibility, transferability, dependability, and confirmability [27]. To establish credibility, we used the technique of prolonged observation, conducting a large volume of semi-structured interviews, many lasting over one hour, over the course of several days at each of the sober houses. We also used triangulation of sources by asking both service users and providers some of the same questions, such as about the daily routine of the sober house, to assess consistency between participants. For establishing dependability, we used the external audit method by asking a member of the study team not involved in the data collection to participate in coding and to engage with the data collectors and other study team members who led the data collection process. Transferability was established through the “audit trail” method [28], which entailed collecting daily reports from data collectors and documenting the data collection process throughout site visit days. Confirmability was established through reflexivity throughout the data collection and analysis processes, which was facilitated through conversations among a large study team that combined American and Tanzanian researchers.

### Ethical considerations of study recruitment, data collection, and analysis

Given the sensitive nature of this study, several steps were taken to ensure the privacy and dignity of study participants. The data collectors for this study were experienced in qualitative research and were instructed to terminate an interview if a study participant showed signs of discomfort or distress. Written informed consent was gathered prior to both the survey and semi-structure interview, and interviews were conducted in a private space. Moreover, data collectors signed a confidentiality agreement prior to data collection and all data were anonymized prior to analysis. The study team collaborated closely with sober house administration prior, during, and after data collection, to ensure that study participants felt respected throughout the process. All data collected through audio recordings and written surveys were uploaded to a secure, password-protected, cloud-based storage system, accessible only by select study team members.

## Results

### Description of study population

Across the four sober houses in our sample, there were 117 service users in total, which included 92 men and 25 women (Table 1). Of the 117 service users, 86 completed a survey (73 males and 13 females) and 38 participated in interview (31 males and 7 females). The primary reasons service users did not complete a survey or interview were that they were sleeping while it was being administered, were out of the house for an appointment, or stated they were too busy with chores. The total service provider population across the four houses was 12 (excluding non-program staff such as cooks, security guards, etc.) and 10 service providers members completed a semi-structured interview (8 from male sober houses, 2 from female sober houses).

**Table 1:** Demographics and personal history (*n* = 86)

**Sex**				
Male	Female			
92 (73.6%)	25 (21.4%)			
**Current Age**				
Median	Range	Standard Deviation		
35.2	19–56	9.85		
**Religion**				
Christianity	Islam	Hinduism	Other/None	No response
51 (60%)	29 (34.12%)	1 (1.18%)	4 (4.71%)	1 (1.2%)
**Highest level of education completed**				
Primary School	Secondary School	Some College	Bachelor’s Degree	Advanced Degree
17 (19.77%)	34 (39.53%)	17 (19.77%)	11 (12.79%)	7 (8.14%)
**Age of first drug use**				
Median	Range	Standard Deviation		
18	10–40	5.33		
**Previous sober house stays**				
Yes	No	No Response		
34 (40%)	51 (60%)	1 (1.2%)		
**HIV diagnosis**				
Yes	No	No Response		
5 (5.8%)	76 (88.4%)	5 (5.8%)		
**Psychiatric diagnosis other than SUD**				
Yes	No	No Response		
23 (26.7%)	56 (65.1%)	7 (8.1%)		

Among service users, the median age at the time of the survey was 35, while the median age of first drug or alcohol usage was 18. Self-identified Christians made up the majority of participants (60%), followed by Muslims (34.12%). Overall, educational attainment was high, with 40.7% of participants reporting at least some college education. The prevalence of HIV among survey participants was 5.8%, slightly higher than the national average of 4.5% [29]. There was also a high co-occurrence of mental illness among service users with more than one-quarter of participants (26.7%) reporting a diagnosis of a mental illness other than SUD. Most participants reported their current sober house stay was their first, but a substantial minority (40%) reported previous treatment in a sober house.

### HIVST and PrEP use, knowledge, and willingness to use

As shown in Table 2, familiarity with and previous experience using HIVST and PrEP was low among participants. While just over half of participants had heard of an HIVST kit (51.2%), less than a third of participants had ever used one (29.1%). Familiarity and experience with PrEP was even lower, as less than 1 in 5 (18.6%) participants reporting familiarity with PrEP or ever having used it. However, willingness to use HIVST and PrEP was high, and over 70% of participants expressing that they would be interested in using HIVST or PrEP if they were made available in the sober house.

**Table 2:** HIVST and PrEP knowledge, history, and willingness to use (*n* = 86)

**Have you ever heard of an HIV self-test kit?**				
Yes	No	No Response		
44 (51.2%)	40 (46.5%)	2 (2.3%)		
**Have you ever heard of PrEP?**				
Yes	No	No Response		
16 (18.6%)	67 (77.9%)	3 (3.5%)		
**Have you ever used an HIV self-test kit?**				
Yes	No	No response		
25 (29.1%)	55 (63.9%)	2 (2.3%)		
**Have you ever used PrEP?**				
Yes	No	No response		
16 (18.6%)	67 (77.9%)	3 (3.5%)		
**Based on the information provided, would you be willing to use an HIVST kit if offered in the sober house?**				
Yes	No	Unsure	No response	
62 (72.1%)	12 (13.9%)	5 (5.8%)	7 (8.1%)	
**Based on the information provided, would you be willing to use PrEP if offered in the sober house?**				
Yes	No	Unsure	I am not eligible due to my HIV status	No response
61 (70.1%)	14 (16.3%)	8 (9.3%)	1 (1.2%)	2 (2.3%)

## Life before the sober house

Exploring service users’ lives before entering the sober house and their decision to enter treatment is critical for understanding their acquisition of and deficiencies in recovery capital, and how recovery capital is built throughout a sober house stay.

### Educational and professional history

Many service users reported that prior to entering the sober house they possessed a high level of personal recovery capital related to their employment and education, particularly before acceleration of their substance use. Related to their educational history, one resident mentioned that sober house service users sed a range of educational levels, stating:

*Their education levels vary…Some here have completed primary school, some finished high school (Form Four and Form Six), and there are those who attended vocational schools and universities* (Male service user, ID: 0019).

Among those who had received higher education, participants mentioned a broad range of academic disciplines. These fields of study included economics, fine arts, computer science, and accounting. Service users reported that they held a wide range of jobs, including consulting, accounting, and teaching. Some participants also reported training in vocational trades, such as automative repair and electrical work. Many also explained that they had run small businesses, either as their primary occupation or for supplemental income. These businesses varied in size but were almost exclusively focused on retail. Service users sold a range of products including cell phones, kerosene, diesel, and used clothing. When discussing how he started his small business, one respondent discussed receiving the help of a business partner, indicating the presence of social recovery capital:

*I used to be a businessperson… I used to sell mattresses… I didn't start from scratch; I worked with someone, and after some time, they advised me to continue on my own. They had already shown me the ropes, so I continued* (Male service user, ID: 0016).

### Perceived effect of substance use on personal and professional trajectories

A common theme among service users was the disruptive effect that substance use had on their employment and income, with participants stating:

*Not long ago, my salary was around $3,500 per month, and then suddenly it disappeared. Life became unstable…. I had to change my children’s school from an expensive one to something more affordable* (Male service user, ID: 0008).

*We are all different, as I mentioned earlier, I struggled to get money, and I started using* [drugs] *when I had my own money. I would withdraw money from my account without keeping track. Eventually, everything I owned was gone. I even sold my car and lost a lot due to my drug use* (Male service user, ID: 0004).

Among younger service users, some discussed the way their addictions interrupted their education, with one service user saying:

*I was attending flight school when things went haywire, and that’s when I ended up in this recovery house… I hadn’t completed it when I returned home to address my issues* (Male service user, ID: 0026).

### Social influences and the decision to seek treatment

In discussions with service users regarding their decision to enter the sober house, many recounted the role of peer and familial influence, indicating a strong presence of social recovery capital in their lives. Many learned of the sober house through a family member, who encouraged them to seek treatment and helped arrange for them to be received into the sober house.

One resident explained:

*I didn’t really know about it* [the sober house]*; it was my younger sister who found out. She was aware of my alcohol problem, and she knew that I could disappear for days. She used to tell me that one day I’d disappear and be missing for three days or even four, and people would think it’s normal, but she realized that it might be because I had problems. So, she brought me here because of my alcohol problem* (Female service user, ID: 0006).

While social recovery capital from supportive family members was essential to many service users’ decision to seek treatment, they also noted the way negative social influences played a role in their substance use:

*To be honest, I’ve been through all of this before and I’ve accepted that these people I drank with are acquaintances, not close friends. Because close friends would care about you. For example, if you’re drinking a lot, they’d tell you it’s enough. These people just let you continue, so I’ve accepted that they’re not close friends. My goal is to reduce my interaction with them because my aim is to recovery* (Male service user, ID: 0002).

While some service users were convinced to enter the sober house by family members, many others reported that they did not enter the sober house by choice and were brought through deception. One resident told his story of entering the sober house:

*When I woke up, I was in a new place, and he* [the manager] *welcomed me as a new member. I was confused and asked, "New member of what?" I wanted to give him a hard time, but he offered me tea instead. I asked him, "Why should I drink tea? Where am I?" He said I should drink the tea, and I realized that I had no idea where I was. Another staff member came and told me that my younger brothers had taken me here and explained that I was now in a sober house. They told me that my brothers had said I needed help and agreed to help me. I was surprised and didn’t know what to think at that moment* (Male service user, ID: 0021).

Related to service users entering through deceptive means, a sober house service provider confirmed these stories, noting that often families felt deceiving their family member was their only option:

*Their families* [service users’] *see their condition and want to help, so they ask us to pick them up because they can’t come on their own. They believe they’re fine and can still use substances… but the things they do, both to their families and the community, are unacceptable. Although they think what they’re doing is fine, it’s actually problematic. So, their families and the community ask us to pick them up because they won’t come on their own* (Service provider in female sober house, ID: 0004).

### Services and programming offered in the house

All participants were asked during interviews to recount a typical day in the sober house, and then were probed by interviewers to expand upon each activity they mentioned. Participants were also asked about services that may be provided to a service user at one point in their sober house treatment period but not in others, such as a health screening or detoxification services. The responses between service users and providers across all 4 sober houses were very similar and service users across houses generally followed a similar schedule. One sober house was distinct in that it was managed by an Emergency Medicine physician, who reported being able to provide more intensive health services than were provided in the other 3 houses, which did not have medical service providers. Service providers were also asked about the cost of treatment. Treatment costs ranged from 160 to 600 USD monthly. This cost includes room and board, as well as the cost of all treatment received in the sober house. However, generally those fees did not include expenses like personal hygiene products, snacks, and medical bills, should a patient require hospitalization or extensive outpatient treatment. The services offered in the 4 sober houses are summarized in Table 3 as told to data collectors during interviews.

**Table 3:** Description of sober house services and programming

	**Description**	**Availability**
Detoxification upon entry	• Dedicated period, usually lasting 5–7 days, where clients in active substance use complete a full detoxification from substances • Clinical oversight from staff member (1 house, male) or in consultation with a clinician by phone (3 houses) • Use of non-narcotic pain medications and rehydration through an IV	All 4 sober houses
Health Screening Before or Immediately Following Entry	• Physical examination of residents either conducted at facility (1 house, male) or external facility in the surrounding community (3 houses) • Can complete before entry if resident uses standardized sober house paperwork for physician to record examination results • Residents receive a series of blood tests to check their overall health, including tests for liver and kidney function, a full blood count, and screening for sexually transmitted infections (STIs) such as hepatitis and HIV • Blood pressure and blood glucose screening	All 4 sober houses
AA/NA Meetings	• Daily, 12-step Alcoholics Anonymous(AA) or Narcotics Anonymous(NA) meetings for all current residents and some residents receiving aftercare	All 4 sober houses
“Daily Feeling Sessions”	• Morning meetings with all residents, led by a staff member where residents express the mental/emotional state they are experiencing in the present moment • Residents are encouraged to provide peer support and encouragement to each other	All 4 sober houses
Recovery Education Sessions	• Daily sessions (often twice daily) where residents learn principles of 12-steps and receive psychosocial education about managing addiction • Led by a staff member or community member who is in recovery, including past residents	All 4 sober houses
Household chores	• Dedicated period (usually lasting 30–60 min) for completion of household tasks among residents including landscaping, food preparation, housecleaning, etc	All 4 sober houses
Ongoing health checks with clinical provider	• Health checks are only performed upon client request, after initial screening generally for acute issues (stomach infection, malaria, etc.) or when client has diagnosed chronic condition requiring ongoing management	All 4 sober houses
Distribution and Management of Medications	• Resident medications are stored in a locked box in staff offices • Daily distribution of medications by a staff member • Ongoing medication management by in-house clinical staff (1 house, male) or through consultation with external clinical staff in surrounding community	All 4 sober houses
Formal life skills training (soap making, baking, etc.)	• Life skills and income generating workshops such as baking, soap and lotion making, vegetable gardening • Offered by staff with relevant skillset, or outside community members	1 sober house (male)
Visits by faith-based groups	• Visits ranging from weekly to monthly from community religious leaders or affiliated volunteers • Conduct Bible study, lead worship services • Sometimes lead other recreational activities such as art projects	3 sober houses (2 male, 1 female)
Aftercare	• Ongoing support to residents after treatment completion and reentry into the community, typically lasting up to one year • Includes attendance at daily AA/NA meetings in the sober house and opportunities for service in the sober house, including assisting in recovery educational sessions • Celebration of sobriety milestones (6 months, one year, etc.)	All 4 sober houses

### Perceptions of and gaps in health service provision

After explaining the services and programming offered in the sober houses, participants were asked about the barriers and facilitators to delivery of these services, perceptions of quality, and where they see gaps in service provision. Related to the health screening that service users receive, service providers universally believed that the screening was important, and that it is mandated by the DCEA. However, service providers explained that fulfillment of the health screening requirements is time-intensive and costly, and that often those costs are covered by the sober house. Some of this cost is derived from the absence of government health facilities in the communities surrounding the sober house, which may be able to provide low- or no-cost health services. One service provider explained:

*For instance, they’ve mandated that every client should undergo specific medical tests. Often, these tests are not available at the nearest hospitals. We end up taking these clients to private facilities, which increases the overall cost. This adds to the financial burden, and we cannot provide these services for free. So, while the government sets regulations, it can be difficult to meet all the requirements* (Service provider in male sober house, ID: 0001).

The costs of health services are also increased by a reliance on external health care providers due to the lack of clinicians on staff at three of the sober houses. One service provider said:

*We have a pharmacist that we collaborate with. We usually call them, and they come here to set up the drip and provide the pain medication* [during detoxification]*. But I continue with the pain medication according to the doctor’s instructions because I have experience now. So, I give it to them based on the doctor’s prescription, but the pharmacist handles the drips* (Service provider in male sober house, ID: 0005).

Many service users confirmed the health check-up process, though several expressed inconsistencies in the process, and some reported that ongoing medical screening for HIV and other diseases was not routine in the sober house:

*Honestly, I was worried at some point, and I went to a lab for an HIV test. Fortunately, I tested negative. But that was a long time ago. I haven’t tested for about eight months* (Male service user, ID: 0003).

Others reported that there were long periods of time between entering the sober house and being brought for their health screening, indicating gaps in service provision:

*No, there were no medical tests when I arrived. However, we recently went for testing about two weeks ago* (Female service user, ID: 0017).

However, like providers, service users placed a high value on the need for close monitoring of their health, particularly for HIV and psychiatric illnesses. One service user added that, in addition to HIV services, there is a need for more education regarding HIV prevention and mental health in the sober house:

*Even regarding HIV education, we need it greatly. Many of us don’t have a complete understanding of that issue. As you can see, there’s also a significant problem with mental health among the residents. Some are using medication, some have quit, and others are still struggling. So, there’s a need for that education too* (Male service user, ID: 0006).

Service providers agreed with the need for more HIV services and education, saying:

*In terms of diseases, many of them engage in high-risk behaviors like unprotected sex and other risky activities while they are using. They often engage in these activities in places where there is a high risk of contracting diseases, including skin diseases* (Service provider in male sober house, ID: 0018).

### Perceptions of and gaps in other service provision

Service users were asked to consider the services they are receiving, the way these services increase their acquisitions of recovery capital, and whether these services were equipping them adequately to remain abstinent from substances after leaving the sober house. On this topic, service users noted the importance of staying busy and reducing idle time after leaving the structured environment of the sober house:

*So, they asked me what causes me to relapse, and I explained that when I go back home, I have nothing to do. They can’t give me a job until they are sure I’m doing well. So, they spend time checking on me and, during that time, I have nothing to do. Most of the time, this is what pushes me back to my old habits because I have nothing else to do* (Male service user, ID: 0003).

Many expressed that they had become well-initiated into the AA/NA program during their time in the sober house. These meetings increase their personal recovery capital by equipping them with strategies to maintain substance use abstinence and increase social recovery capital by linking them to a community of supportive peers. One resident identified attending AA/NA meetings and finding steady employment as his primary strategies for maintaining recovery:

*The most important thing for me is to stick to the NA program, and secondly, to engage in business or employment. But the key is to understand that work alone is not the solution. Without participating in this community (NA)-- work alone won’t help me. Right now, my therapy or medicine is attending meetings and engaging with this community because they understand how we are and know how to help us stay clean* (Male service user, ID: 0009).

When discussing employment prospects after leaving the sober house, service users noted the toll that their substance use had taken on their social recovery capital—primarily due to high levels of community stigma towards people in recovery. One resident noted community stigma towards people with a history of substance use, saying:

*We’d use* [substances] *then look for ways to get more money to continue using. This led to a loss of trust from people towards us. For instance, before selling fruits, I had worked at a motorcycle spare parts shop. I was fired because I used drugs and stole things. So, when people see me going back to that shop, they might tell me to come back tomorrow. As I leave, another person comes to ask why I was there, and they might say I’ve come to apply for a job, but they’d be told I’m a thief. So, people’s perception of my past affects my job search* (Male service user, ID: 0004).

A sober house service provider echoed a loss of trust that many people with SUD face in their communities, and explained the way the absence of community recovery capital impacts their employment prospects:

*First, people who recover from addiction lose confidence after recovering. They tend to think ‘How do people think of me? Like I am not trusted so they cannot give me a job because they know my past, that I was drunk or a drug addict. This makes us feel ashamed to apply for jobs or makes it harder to get a job in places where they know us. There is a saying “Mwendawazimu haponi.” That means a person with mental illness is never healed. But I believe they can get better* (Service provider in female sober house, ID: 0026).

To try to encourage self-sufficiency and limit lack of activity, one service provider mentioned providing some limited entrepreneurship and life skills training that may be leveraged for income generation or as a hobby after completing treatment. This service provider explained:

*Sometimes we bring entrepreneurship trainers here at the center to train residents in different skills. For example, we bring trainers who teach residents how to make soap where they learn how to make liquid soaps and bar soaps like these ones here. We also bring artists who train residents in artistic works such as drawing. We believe that the skills that the residents acquire here at the center will be their starting point when they go back to the community while waiting for official jobs rather than just staying idle* (Service provider in male sober house, ID: 0018).

Overall, participants expressed positive perceptions of the sober house venue, while acknowledging areas for improvement. One service provider reflected on her own journey through treatment in a sober house, and eventual employment as a peer counselor:

*We tend to judge without knowing the underlying reasons. You see, everyone has their story, and I have learned through this work that love and understanding can change people. I was shown love, taught that change is possible, and that there’s a way out* (Service provider in female sober house, 0004).

## Discussion

The data indicate both significant strengths and unmet needs within sober house treatment programs. Service users enter sober houses under vastly different circumstances, with some admitted through coercive means. Commitment to one’s substance use recovery is part of personal recovery capital and was reported to often increase over a resident’s time in the sober house. However, this still presents a challenge early on in a sober house stay for those who did not voluntarily seek treatment, and studies show that involuntary substance use treatment is generally less effective, can be harmful to one’s treatment outcomes, and is largely not supported by people with a SUD [30–32].

Service users included in the study possess diverse educational and professional backgrounds, which overall indicate a high degree of personal recovery capital. Over 40% of survey participants reported at least some university education (including completion of a certificate program). This is significant given the relatively low levels of educational attainment among Tanzanian adults. As of 2022, only 19.2% of Tanzanians over age 15 had completed secondary school, 1.9% had a university degree, and 2.5% report receiving vocational training [33]. While a more comprehensive study would be needed to examine overall rates of educational attainment among sober house service users nationwide, these preliminary data indicate that they likely possess higher levels of education than the general population of Tanzania. This may be due to factors influencing the composition of sober house populations including younger age, the location of sober houses in urban areas, and the role of affordability in who can access treatment. The median income in Tanzania is just 702 USD per year, or 58 USD per month, making sober house treatment highly cost prohibitive for many [34]. In Tanzania, like many other countries in SSA, cost is a significant barrier to residential treatment for SUD, as most residential treatment programs only accept private pay [35].

Still, there is significant socioeconomic heterogeneity among sober house service users. As several participants confirmed during their interviews, the range of educational and professional experiences of sober house service users is vast, and survey data supported this observation as nearly 1 in 5 participants (19.77%) had only received a primary school level of education. The diverse socioeconomic characteristics of service users is consistent with studies conducted throughout the world which show that while certain socioeconomic factors may offer some protection against SUD, the individuals affected still come from all levels of education and wealth [36, 37]. Service users’ diverse range of educational and professional backgrounds is a necessary consideration for future studies that seek to augment sober house services.

After completing treatment, sober house service users experience diminished social and community recovery capital, namely due to doubts among community members and prospective employers about their ability to remain abstinent from drugs and alcohol. Generally, service users expressed that they plan to return to the communities in which they lived prior to entering the sober house. While this may be helpful to service users by placing them close to family and social support, it also leaves them vulnerable to facing community stigma from those who knew them during periods of active substance use. Participants expressed that reentry into the workforce is difficult for service users given their sustained break in employment while receiving treatment, as well as difficulty in securing start-up capital if they would like to start a business. Other studies conducted among sober house service users in Tanzania have shown that community stigma is a major driving force in substance use recurrence and eventual readmission to the sober house [19].

Service providers expressed a strong commitment to ensuring adequate health services for residents while receiving treatment but identified cost of treatment and a lack of clinical providers working in the sober house as barriers to providing comprehensive healthcare. Service users expressed enthusiasm about enhancing the HIV care available to them in the sober house, including the provision of HIV self-testing kits and PrEP, both in written surveys and during semi-structured interviews. The self-reported HIV prevalence rate of sober house services users was dramatically lower than national prevalence estimates among PWUD (5.8% vs 36%) which may indicate that sober house users benefit from certain protective factors against HIV that are not experienced by the broader population of PWUD in Tanzania. One recent study of substance use treatment programs found that HIV testing and PrEP can be successfully integrated in these settings, and that leadership engagement is key determinant of successful integration [38]. Service providers in our sample showed high levels of enthusiasm to expanding medical care to service users, but further research is needed to understand potential barriers and facilitators of integrated HIV services in sober houses.

Relatedly, while many residents are taken for health screenings prior to or immediately following their entry into the sober house, only one of the houses in the study sample had a full-time clinical provider on staff. One strategy for addressing cost and the lack of clinical providers working in the sober house could include leveraging the Nurse-Initiated Management of ART (NIMART) program in Tanzania. Established in 2018, NIMART allows nurses in Tanzania to order HIV tests, prescribe ART and PrEP, and manage ongoing testing and medication management for patients [39]. NIMART programs throughout SSA have been shown to be effective strategies to increase uptake of HIV testing and treatment [40] and the incorporation of NIMART into Tanzanian sober houses would be a novel extension of this program.

Studies concerning residential substance use treatment programs in SSA are limited and have largely been confined to just a couple of countries, namely South Africa and Nigeria [35]. An even smaller proportion of these studies have sought to garner service user and provider feedback on the treatment programs, perceptions of the quality of services, and unmet needs of both those receiving treatment and their providers [35, 41, 42]. An enhanced understanding of the unmet needs of services users and providers is essential in identifying where opportunities for interventional or implementation research lie within the largely unexplored sober house setting.

### Limitations

There are a couple of limitations to this study worth noting. The low proportion of female participants in the study sample is indicative of the underrepresentation of women in Tanzanian sober houses but poses a challenge in determining if the results are fully transferable to women. The location of all four sober houses in Dar es Salaam may limit the transferability of results for the development of an interventional study if there are significant variations in sober house operations and populations in other parts of Tanzania.

## Conclusion

Guided by our three study questions, we explored the services offered in sober houses and the perceptions of service users and providers towards those services. We found generally positive perceptions of the services offered, and that sober houses provide much of the programming that is considered essential in substance use treatment programs including peer support, 12-step meetings, and psychoeducation [43–45]. However, employment preparation services and status-neutral HIV care, which have both been integrated extensively in other substance use treatment settings, are largely absent from sober houses [38, 46]. These unmet needs hinder service users’ recovery after completing treatment, particularly when using the definition of recovery articulated by the Betty Ford Institute which includes not only sobriety, but also personal health [47]. Interventions that address these unmet needs may be feasible in sober houses, but further research is needed to identify appropriate interventions as well as barriers and facilitators to implementation in this unique treatment context.

## Supplementary Information

Supplementary Material 1.

Supplementary Material 2.

Supplementary Material 3.

## Data Availability

All data generated or analyzed during this study are included in this published article [and its supplementary information files].
