# Supplementary material for: Exploring perceptions of the services offered in Tanzanian sober houses: a mixed- methods study among service users and providers
Source: BMC Health Serv Res. 2025 Feb 14;25:253. doi: 10.1186/s12913-025-12384-7 (PMC11829410; doi:10.1186/s12913-025-12384-7)
Supplement: Supplementary file 1 — Supplementary Material 1. [file 12913_2025_12384_MOESM1_ESM.docx]

**Personal Information**

1. What is your age (in years)?

________________________________________________________________________

1. What is your tribe?

________________________________________________________________________

1. What is your religion?
   1. Christianity
   2. Islam
   3. Hinduism
   4. Other: ___________________________________
   5. None
2. What is your place of residence?

________________________________________________________________________

1. What is your highest education level completed?
   1. Primary School
   2. Secondary School
   3. Some College
   4. Bachelor's Degree
   5. Graduate/Professional Degree

**Personal History**

1. How long have you been in the sober house (in weeks)?
2. Have you ever stayed in a sober house before now?
   1. Yes
   2. No
3. If yes, how many additional times have you stayed in a sober house before now?
   1. 1
   2. 2
   3. 3
   4. More than 3
4. How old were you when you first used drugs or alcohol?

___________________________________________________________________________

1. What was the first type of drug or alcohol you used?
2. Alcohol
3. Heroin
4. Cocaine
5. Marijuana
6. Methamphetamine
7. Prescription pills
8. Other:____________________________________________
9. Is there one substance you use consider your primary substance of choice? If yes, please answer below. If not, please skip to Question 12.
10. Alcohol
11. Heroin
12. Cocaine
13. Marijuana
14. Methamphetamine
15. Prescription pills
16. Other:__________________________________________
17. In addition to your drug of choice, what other drugs do you use?
    1. Alcohol
    2. Heroin
    3. Cocaine
    4. Marijuana
    5. Methamphetamine
    6. Prescription pills
    7. Other:__________________________________________
    8. None
18. How long were you using drugs or alcohol before entering the sober house? If you have been treated in a sober house multiple times, please state the amount of time before this treatment episode.

_____________________________________________________________________________

**Medical**

1. Have you ever been tested for HIV?
   1. Yes
   2. No
   3. Unsure
2. Have you ever been diagnosed with HIV?
   1. Yes
   2. No
   3. Unsure
3. On average, how often would you say you are tested for HIV?
   1. 4 times a year or more
   2. 3 times per year
   3. 2 times per year
   4. 1 time per year
   5. Less than 1 time per year
4. Do you know what an HIV self-testing kit is?
5. Yes
6. No
7. Unsure
8. Have you ever used a self-test for HIV?
   1. Yes
   2. No
   3. Unsure
9. Would you be willing to use one?
   1. Yes
   2. No
   3. Unsure
10. Have you ever been diagnosed with any form of Hepatitis?
    1. Yes
    2. No
    3. Unsure
11. On average, how often would you say you are tested for Hepatitis?
    1. 4 times a year or more
    2. 3 times per year
    3. 2 times per year
    4. 1 time per year
    5. Less than 1 time per year
12. Have you ever been diagnosed with a mental health condition?
    1. Yes
    2. No
    3. Unsure
13. If yes, which mental health condition have you been diagnosed with?
    1. Depression
    2. Anxiety
    3. Bipolar disorder
    4. Schizophrenia
    5. Other____________________________________________________________

1. Have you ever used a needle for injecting drugs?
   1. Yes
   2. No
   3. Unsure
2. During sex, do you use a condom:
   1. Always
   2. Sometimes
   3. Rarely
   4. Never
