# Supplementary material for: Exploring perceptions of the services offered in Tanzanian sober houses: a mixed- methods study among service users and providers
Source: BMC Health Serv Res. 2025 Feb 14;25:253. doi: 10.1186/s12913-025-12384-7 (PMC11829410; doi:10.1186/s12913-025-12384-7)
Supplement: Supplementary file 2 — Supplementary Material 2. [file 12913_2025_12384_MOESM2_ESM.docx]

| **Sober House Resident Interview Guide** | |
| --- | --- |
| *Thank you for taking the time to speak with me today. I want to remind you that anything we discuss today will be private and will not be shared with anyone, including other residents or staff. If there are any questions that you would rather not answer, or you are unable to answer, please tell me and we will skip that question. If any of the questions do not make sense, please ask for clarification. The purpose of this interview is to understand what motivated you to enter the sober house, what your experiences have been like while staying here, and what improvements could be made to better meet your needs in the house and in the community.* | |
| **Main Question** | **Probes** |
| Can you please tell me your age, tribe, and religion? |  |
| Where did you grow up? | If somewhere else, how long have you lived in the area? |
| Please tell me about your life before joining the sober house. | Are you married?  Do you have any children?  What is your occupation?  -Do you believe you’ll be able to go back to your job once you finish treatment? |
| How long have you been in the sober house? |  |
| How long do you plan to stay in the sober house? |  |
| Have you ever stayed in a sober house before now? | If yes, when?  What was that experience like?  Did you feel it helped you in your recovery? |
| **Motivations to Enter Sober House** | |
| *Thanks for sharing that with me. I want to discuss a little about how you ended up at this sober house, at this time in your life, and if anyone helped you make the decision to come here.* | |
| **Main Question** | **Probes** |
| What was your personal experience of the process for joining this sober house? | Did you have other alternative choices for sober houses?  What made you choose this one?  - To what extent was cost considered?  - How much are you paying here? Is someone helping you pay for the fees to be here?  How difficult was deciding to join the sober house for you?   - Did anyone encourage or discourage you from entering? If someone did encourage you, how did they do it? (providing information, financial help, visiting with you) If discourage, how so? |
| Please tell me a little about your personal experiences with substance use. | What was the substance you first used?   - Is it the same drug/alcohol that led you to come to the sober house? |
| Can you please tell me how old you were when you started to use drugs or alcohol? | To the best of your memory, what motivated you to use drugs/alcohol? Influence of friends/family? Stress? Curiosity?  How long have you been using this alcohol/drug?   - Was it consistent (daily)? Off and on?   - **If opiates—**what method did you typically use for taking it (smoking, injection, snorting?   How much would you estimate you spent per week on drugs or alcohol?  Had your parents/relatives ever used alcohol/drug when you were growing? If yes, to what extent? |
| Before entering the sober house, did you try to stop using drugs or alcohol? | If yes, what methods did you try?  -Did you try initially cutting down on usage? Or stopping all at once?  How has your experience in the sober house compared to your previous attempts to stop using drugs? |
| What is your experience in seeking treatment of alcohol/drug use? | Can you tell me what, if anything, you know about Medication Assisted Therapy (MAT)?   - Have you ever received any medication assisted treatment (MAT) such as methadone? -If so, where and for how long?   Did you ever attend AA meetings in the community prior to entering the sober house? Other outpatient care?  Have you talked to your doctor about coming to the sober house? What were their thoughts and treatment recommendations? |
| **Experience in Sober House** | |
| *I now want to discuss your time in the sober house, thinking back to when you first came here, up until our discussion right now.* | |
| **Main Question** | **Probes** |
| Please tell me about your experience of staying in this sober house. | What was the reception process like prior to and after arrival?  -Did you receive an orientation? Complete a health screening? Did you help set up a personal goal plan?  Can you tell me about rules and regulations in place at this sober house and what happens if someone breaks these rules? |
| In general, how would you say you feel about being in the sober house? | Do you feel supported? Anxious? Stressed? |
| What are the health services provided in the sober house? | What, if any, mental health services are being offered at this facility?  Were you asked about your health problems, including mental health conditions, upon arrival?  Were you screened for diseases such as HIV, Hepatitis or other STIs in the house or prior to entering?  What was your general health status when you entered this sober house?  -Were you on any form of treatment or medication?  -How do you manage the treatment in this sober house? |
| What other services do you wish were offered in the sober house? | Employment training? Entrepreneurship? Employment skill building? |
| What does a typical day look like for you here? | What activities do you participate in?  -Doing chores?  -Group meetings?  -Individual or group counseling? Psychoeducation (learning about addiction)?  -Recreation/sports?  Are you ever allowed to leave the sober house while participating in the program?  -Can you have visitors?  -Can you make phone calls?  What activities do you believe help you the most in your recovery? Why? |
| At any point is your family involved in the treatment you receive in the sober house? | If yes, how? |
| In your experience, can you tell me about whether you believe drugs and alcohol are available and used in the sober house? | Have you ever used drugs or alcohol while living in the sober house?  - If yes, how did you get it?  -Where did you use it?  Do staff ever search your belongings to try to prevent drugs in the sober house? If yes, how often? |
| In general, would you say you feel safe or unsafe in the sober house? | Have you ever witnessed violence?  -If yes, what was the context? |
| **HIVST Risk Factors, Knowledge, and Interest** | |
| I want to ask about a few personal behaviors that can have an impact on your health and recovery. | How common is needle sharing for drug injections?  Have you ever engaged in needle sharing?  -If yes, what was the context?  -Did you do anything, like needle cleaning, to try to lower the risk?  -How do you obtain new needles when you need them? Is cost a barrier?  How do you protect yourself from STIs, such as HIV, during sexual intercourse?  -How often would you estimate you use condoms during sexual intercourse? **Sometimes/Always/Never?**  -If never, what is your reason for not using them? |
| Can you tell me about your experiences with HIV testing? | Have you ever been tested for HIV?  -If yes, when and where?  - What was the result?  Have you been tested in the sober house?  If you received a positive result, did you get a confirmatory blood test in a clinic?   - Did you begin ART? If not, why not? - How do you manage your HIV in the sober house?   If results were negative what measures are you taking to protect yourself from HIV?   - Have you heard of PrEP? Are you taking it? |
| Can you tell me what you know about HIV self-testing kits? | Have you heard of the kit before? If so, where?  Have you ever used a self-testing kit?  -If yes, how did you get it? What motivated you to access and use the kit? |
| **If they have not heard of HIVST:** *HIV self-tests are a test that anyone can perform on themselves to check for HIV. They do not require a doctor or other medical professional to perform the test. The test uses a small swab that you put in your mouth to collect saliva, and then a device that tests that saliva. Usually, people get results within 20 minutes. Those getting a positive result then should go to a clinic to get a blood test to confirm their HIV status, and begin taking medication.* | |
| With this explanation in mind, what questions or concerns would you have about using HIVST in the sober house? | How do you think others in the sober house would feel about using HIVST? |
| Would you be interested in using an HIV self-test kit? | If yes, why? If no, why not? |
| **Community-Based Services** | |
| Now, I want you to think about your life after leaving the sober house. What things do you anticipate will help you to stay sober? | What factors do you anticipate make it difficult for you to stay sober?  -Cravings? Influence from family members or peers?  What barriers do you foresee as interfering with you getting the services you need to help you stay sober?  -Availability, time, cost?  Can you tell me about the support system you have to turn to for help after you leave the sober house? |
| What about staying at the sober house has helped? | What things do you think you would change about the sober house to make your stay more helpful to you?  What would you say are your primary motivations for staying sober? |
