# Supplementary material for: Exploring perceptions of the services offered in Tanzanian sober houses: a mixed- methods study among service users and providers
Source: BMC Health Serv Res. 2025 Feb 14;25:253. doi: 10.1186/s12913-025-12384-7 (PMC11829410; doi:10.1186/s12913-025-12384-7)
Supplement: Supplementary file 3 — Supplementary Material 3. [file 12913_2025_12384_MOESM3_ESM.docx]

| **Sober House Staff Interview Guide** | |
| --- | --- |
| *Thank you for taking the time to speak with me today. I want to remind you that anything we discuss today will be private and will not be shared with anyone, including sober house residents or staff. If there are any questions that you would rather not answer or you are unable to answer, please tell me and we will skip that question. If any of the questions do not make sense, please ask for clarification. The purpose of this interview is to know how sober houses operate, describe the services offered here, and understand some of the challenges in the important work of helping people recover from drug and alcohol dependence.* | |
| **Main Question** | **Probes** |
| First, please tell me your age, religion, tribe, and highest level of education completed. |  |
| Please describe for me your experience of working in the sober house. | What are your main roles and responsibilities?  What is the typical daily routine at the sober house?  How long have you worked in the sober house?  What motivated you to work in the sober house?  Have you worked in any other sober house?  -What differences do you see between there and here?  Does anyone visit here from the community/other healthcare institutions for work-related business?  -What are their roles? |
| What do you like about your work here? | Did you receive any training before starting your work here?  -Have you ever received any training related to harm reduction strategies for people who use drugs?  Do you feel that the sober house is adequately staffed?  -If not, what type of staff members do you feel are needed? |
| Can you describe for me the typical schedules of employees here? | Is there a staff member at the house at all times?  - If so, what is their role? Guard, monitor, etc.? |
| **Sober House Operations** | |
| *Thanks for sharing that with me. I now want to discuss some of the operations of the sober house.* | |
| **Main Question** | **Probes** |
| Please tell me about the admission process for residents in the sober house. | How does the community typically hear about the sober house?  Do you advertise? Word of mouth or referrals?  Do you collaborate with any medical facilities in the area for referrals? |
| How many residents can you accommodate in the sober house? |  |
| What is the admission process for the new residents - case notes, orientations, health history and screening etc.? | Are residents ever tested for diseases in the sober house such as HIV or Hepatitis?  Are some potential residents not allowed to enter the sober house because of complex health issues?  -If yes, where are they referred for additional services? |
| How long do the residents stay during treatment at the sober house? | Do most residents typically stay the same length of time? |
| How much do residents pay while staying here, and how do they pay? | What happens for those who can’t afford a payment while staying here?  Do all residents pay the same amount? |
| Please tell me how you monitor the treatment progress of the residents. | What treatment goals do you set with the residents?  How do you assess the progress of recovery for residents?  What form of record keeping is in place in the sober house?  Do you ever collect feedback on client satisfaction?  -If yes, what is the process? (Frequency, method, etc.) |
| **Sober House Services** | |
| *I now want to discuss the services offered in the sober houses, and any treatment philosophies that may inform the delivery of those services.* | |
| **Main Question** | **Probes** |
| Can you describe for me what a typical day looks like for a resident in a sober house? | Would you say residents follow a similar schedule each day?  Are residents allowed to leave the sober house?  -If yes, what are the restrictions when they leave?  What do residents typically do with their free time during a typical day?  -Are there books, exercise equipment, television, etc. available for recreation? |
| What treatment programs do you offer to residents of the sober house? | Can you tell me about AA/NA meetings in the house?  -If these occur, how often? Who leads them? How long do they last?  What, if any, educational sessions or lectures are offered in the sober house about managing addiction?  Can you describe for me the interactions residents ever have with a mental health clinician or psychologist?  Are clients encouraged to engage in any spiritual practices such as prayer or meditation?  -Do faith leaders ever visit the sober house?  Do residents here ever receive Medication Assisted Treatment (MAT) for their treatment?  Can you describe for me any philosophy of recovery used by the sober house, such as a 12-step model?  What happens if a resident gets sick while staying in the sober house?  -Are they taken to a clinic? Does a nurse or doctor come to the sober house?  -Has a resident over overdosed? How is the staff prepared to respond? |
| Are there services you feel should be available in the sober house, but are not currently? | If so, which? (Health, skill-building, entrepreneurship, etc.) |
| How are residents’ families involved in treatment? | Are there days when family can visit?  Do families receive any education about supporting someone with substance use disorder? |
| How does the government influence the operation of the sober houses? | Governing rules and regulations?  -Services that must be offered?  -Limits on capacity?  -Staff to resident ratio?  In your view, does this sober house adhere to all government requirements? Why or why not? |
| **Evaluating Sober House Services** | |
| *I now want to talk about your opinions on the services offered to residents, and how you measure the success of residents.* | |
| **Main Question** | **Probes** |
| Can you please share with me your opinion on residents’ life after discharge from the sober house? | How do you evaluate if residents are ready to leave?  In general, do you believe the sober house helps people stay sober after they leave?  How do you follow up with residents after they leave? Is this done formally or informally?  What support do you believe is available in the community to help residents stay sober once they leave the house? Do you provide them with links to any facility?  How often do you have repeat clients?  -What, if anything, is done differently at a person's second admission into the sober house?  In what ways do you make use of past residents of the sober house to help the current residents?  -Are they volunteering? If yes, in what capacity? |
| What do you think you do well at the sober house? | What do you think could be improved?  What has held back the implementation of these improvements? |
| What issues would make you recommend or not recommend this sober house to one of your loved ones to this sober house if they were struggling with addiction? | Do you think the clients who come to the sober house here are likely to be able to abstain from drugs in the community? |
